# Essential and Toxic Metals in Oral Fluid–a Potential Role in the Diagnosis of Periodontal Diseases

**DOI:** 10.1007/s12011-016-0660-0

**Published:** 2016-03-04

**Authors:** Malgorzata Herman, Magdalena Golasik, Wojciech Piekoszewski, Stanislaw Walas, Marta Napierala, Marzena Wyganowska-Swiatkowska, Anna Kurhanska-Flisykowska, Anna Wozniak, Ewa Florek

**Affiliations:** 1 Department of Analytical Chemistry, Faculty of Chemistry, Jagiellonian University, 3 Ingardena Street, 30-060 Krakow, Poland; 2 Laboratory of High Resolution Mass Spectrometry, Regional Laboratory of Physicochemical Analysis and Structural Research, Faculty of Chemistry, Jagiellonian University, 3 Ingardena Street, 30-060 Krakow, Poland; 3 Laboratory of Environmental Research, Department of Toxicology, Faculty of Pharmacy, Poznan University of Medical Sciences, 30 Dojazd Street, 60-631 Poznan, Poland; 4 Department of Conservative Dentistry and Periodontology, Poznan University of Medical Sciences, 60-820 Poznan, Poland

**Keywords:** Oral fluid, Metals, ICP, Periodontal disease

## Abstract

Recently, many studies have investigated the relationship between the level of metals in the body and various diseases. The objective of this study was to examine any possible influence of periodontal disease upon the concentration of metals in oral fluid and blood and to explore the usability of applying cluster analysis coupled with the analysis of selected elements in oral fluid, calcium (Ca), copper (Cu), iron (Fe), magnesium (Mg), manganese (Mn), zinc (Zn), cadmium (Cd) and lead (Pb), for effectively distinguishing people affected by periodontitis from healthy individuals. The quantification of eight metals in oral fluid and blood samples was performed by two inductively coupled plasma techniques–inductively coupled plasma mass spectrometry (ICP-MS) and inductively coupled plasma optical emission spectrometry (ICP-OES). Most of the examined elements were detected at elevated concentration in the oral fluid of periodontal patients. However, the differences were statistically significant in the case of three metals: Cu, Mg and Mn (*p* < 0.05). Approximately, fivefold increase in the concentration of Cu, threefold-elevated levels of Mn and a twofold increase in the concentration of Mg were found in the oral fluid of the periodontal patients compared to the controls. Cluster analysis confirmed the statistical significance of the differences in the level of metals in the oral fluid between the two groups in most cases, plus enabled the correct classification of the subjects into patients and controls. The relationship between concentrations of metals and periodontal disease may in the future serve to prevent the development of such disease.

## Introduction

Measurements of the concentration of various substances in the blood and urine form the basis of diagnostic tests and various scientific investigations. In recent years, researchers have been looking for other biological materials which have as much informative value as blood, but are cheaper and less troublesome in their collection. Examples of these types of materials are nails, hair [1] and oral fluid (saliva) [2]. However, before it can be used to assess the state of human health, it is necessary to verify that the concentration of the test specimen in the oral fluid is correlated with its concentration in blood.

The determination of metals in biological fluids causes many problems. First of all, this type of material is characterized by a complex matrix, which may lead to numerous interferences during analysis. Moreover, many metals are present in biological materials at such low concentrations that highly sensitive analytical methods are required for their quantification [3].

Elemental analysis of biological samples mainly involves spectroscopic methods among which most commonly used are inductively coupled plasma optical emission spectroscopy (ICP-OES) and inductively coupled plasma mass spectrometry (ICP-MS). These techniques permit the carrying out of the simultaneous determination of numerous elements, and they also have good metrological parameters, such as low detection limits [3, 4].

Mineral deficiencies cause specific human body dysfunctions [5]. At the same time, the accumulation of toxic or essential metals may also lead to serious health problems or even to death [6, 7]. Recently, many studies have investigated the relationship between the level of metals in the body and various diseases, as well as the mutual interactions between elements [8–12]. Most of the relevant publications are concerned with the relationship between the content of microelements in the body and the incidence of cancer [8, 11–13]. Other diseases, such as cardiovascular disease [9] and hepatitis C [10] have also been considered in this context.

Periodontal diseases are mainly chronic inflammatory conditions of the tissues surrounding, supporting and protecting the teeth. Severe periodontitis can lead to the loosening and loss of teeth [14]. The weakening of the body’s defence mechanisms, thereby increasing the susceptibility to bacterial infection of the periodontal tissues, is dependent on many factors. These can be divided into two group: determinants over which man has no control and the risk factors that increase susceptibility to periodontal disease. Factors such as genetics, sex, race, age, and socioeconomic status belong to the first group, while the second one includes smoking, poor oral hygiene, hormones, improper diet, taking certain drugs, stress and numerous diseases, such as diabetes, osteoporosis and AIDS. Periodontal disease is widespread among the human population and is considered a social disease [15, 16].

Due to high prevalence of periodontal diseases, the identification of biomarkers that can help in clinical evaluation of periodontal status is important. Therefore, many investigators have tried to identify markers specific to periodontitis. Traditionally, diagnosis was based on the measurement of specific biochemical parameters in blood or gingival crevicular fluid, but lately saliva has become a biological material of choice [17]. Among many recognized salivary biomarkers are cytokines, matrix metalloproteinases (MMP)-8 and (MMP)-9 and microbial periodontal pathogens (e.g. *Prevotella intermedia*, *Porphyromonas gingivalis*) [17–19]. Saliva also allows to identify stages of periodontal disease [18]. Considering many biological functions of metals, the examination of the elemental profile during periodontal disease and the identification of changes in the level of metals seem to be interesting and have potential diagnostic value.

The objective of this investigation was to study the usefulness of available methodology to determine the concentration of selected metals in the oral fluid and blood of patients with periodontitis and then compare them with those of healthy controls, by two techniques: ICP-MS and ICP-OES. The possible influence of periodontal disease upon the concentration of metals, calcium (Ca), copper (Cu), iron (Fe), magnesium (Mg), manganese (Mn), zinc (Zn), cadmium (Cd) and lead (Pb), in oral fluid and blood was examined. An attempt was also made to see if chemometric methods could distinguish people with periodontitis from healthy ones on the basis of the elemental levels in two biological materials.

## Materials and Methods

### Subjects

A total of 31 non-smoking patients (17 females, 14 males) diagnosed with periodontal disease were included in the present study. The patients’ mean age was 34.5 ± 10.4 years. Diagnosis of each case was carried out by medical experts, according to clinical symptoms and the Community Periodontal Index of Treatment Needs (CPITN). Subjects were selected from patients of the Department of Conservative Dentistry and Periodontology, Poznan University of Medical Sciences, Poland. The exclusion criteria included the following: clinically significant and diagnosed illness, alcohol and drug abuse and pregnancy. The gender- and age-matched control group consisted of 29 non-smoking volunteers (19 females and 10 males, with a mean age of 31.8 ± 16.8 years) with healthy periodontal tissues (confirmed by dentists) and no known illnesses. All of them live in Poznan or cities of above 100,000 citizens. None of the subjects or controls was occupationally exposed to metals, and dietary supplements were not used by them. They had a typical Polish diet, mainly characterized by a high consumption of meat (pork, chicken) and dairy products, and a low consumption of whole grain products, seafood, fresh vegetables and fruits. Informed written consent was obtained from each participant prior to commencement of the study. Each subject agreed to fill in a questionnaire recording age, gender and smoking habits.

All procedures performed in the study were in accordance with the ethical standards of the institutional and national research committee and with the World Medical Association Declaration of Helsinki (version 2002). Ethical approval (Protocol Number nr 670/08) for the study was obtained from the Bioethics Committee of the Poznan University of Medical Sciences, Poland.

### Sample Collection

The oral cavity was rinsed with deionized water immediately before the oral fluid collection. About 3 mL of unstimulated oral fluid was collected early in the morning, directly into special vessels (Salivettes). Samples of oral fluid were centrifuged at 4000 rpm (10 min). The blood samples (about 8 mL) were withdrawn from a forearm vein, using a stainless-steel needle surrounded by an inert plastic cannula, and transferred to plastic tubes without anticoagulant. Before digestion, all samples were stored at −80 °C.

### Instrumentation and Reagents

A microwave digestion system (Multiwave 3000, Anton Paar, Austria) equipped with eight high-pressure vessels was employed for the decomposition of samples. The Optima 2100 DV (Perkin-Elmer, USA) Inductively Coupled Plasma Optical Emission Spectrometer (ICP-OES), equipped with axially viewed plasma, was used in the determination of Ca and Mg at a wavelengths of *λ*_Ca_ = 317.933 nm and *λ*_Mg_ = 422.673 nm. All other measurements were carried out using the Elan DRC-e (Perkin-Elmer, USA) Inductively Coupled Plasma Mass Spectrometer (ICP-MS). Mn, Fe, Cu, Zn, Cd and Pb were measured using masses of 55, 57, 65, 66, 111 and 208, respectively. The ICP methods were optimized and validated before analysis of the clinical samples. The applied analytical methods enabled the determination of each selected element with satisfactory accuracy (the relative systematic error had a value between 0.1 % for Fe and 8.89 % for Mn), good precision (expressed by coefficient of variation in terms of repeatability was in range from 0.34 % for Ca to 2.74 % for Mn) and low limits of detection (values ranged from 0.007 μg L^−1^ for Pb to 0.21 μg L^−1^ for Fe). The measured concentrations of metals of the reference material were in good agreement with the certified values. Detailed protocols of the measurement procedures were described previously [20]. Argon (Linde, Poland), with a purity of 99.999 %, was used for plasma generation, nebulization and as auxiliary gas.

All reagents used in this study were of analytical-reagent grade or the highest purity available. Ultrapure deionized water (20 MΩ), obtained from WG-HLP deionizing system (Wigo, Poland), was used throughout. Multi-element ICP-MS Calibration Std 3 (containing 10 μg mL^−1^ of each analyte in 5 % HNO_3_, Perkin-Elmer Pure Plus, USA) and Multi-element Standard Solution VI (containing 1000 mg L^−1^ of each analyte, Merck, Darmstadt, Germany) were used for the calibration and control of all the analytical processes. The daily test was performed with the use of Smart Tune Solution Std ELAN & DRC-e (Perkin-Elmer, USA). Certified reference material for blood (Seronorm Trace Elements in Whole Blood, L-2; SERO AS, Billingstad, Norway) was used for the accuracy evaluation. Certified reference material for oral fluid was not commercially available.

### Preparation of Samples

The acid digestion procedure was carried out using a microwave digestion system. Temperature and pressure were controlled during a heating programme with the maximum pressure and temperature of 60 bar and 242 °C, respectively. The blood and oral fluid samples (0.5 mL) from each subject, four replicate samples of certified reference material and a blank sample were directly introduced into digestion vessels and 6 mL of concentrated HNO_3_ was added. The digested samples were transferred into 10-mL volumetric flasks and diluted with deionized water. The sample solutions were stored in plastic vials at 4 °C.

### Statistical Analysis

The normality of the dataset was verified by the Kolmogorov–Smirnov (with Lilliefors correction) and the Shapiro–Wilk tests (*p* < 0.05). In the next step, a basic data analysis was performed to obtain the mean values, median, range and standard deviation. Outlying results were identified by Grubb’s test and omitted in further calculations.

Differences in the concentration of the various metals in oral fluid and blood between the different groups (patients vs. healthy volunteers and men vs. women) were evaluated using a two-sided Mann–Whitney test with a 0.05 significance level. Prior to that, the equality of variance was tested using the Snedecor *F* test (*P* = 95 %). In order to analyse, confirm and visualize the relationship among metals, a Pearson’s (control group) or Spearman’s (patients) correlation analysis was applied to the dataset as well as a principal component analysis (PCA), which allows reduction of the number of observed variables, without much loss of information. The data were normalized before analysis.

The last step was to classify oral fluid samples based on their trace elements concentration by using hierarchical cluster analysis (HCA). Ward’s hierarchical method of agglomeration and the squared Euclidean distance as a measure of the distance between the observations were applied. The cases included in cluster analysis were randomly selected from the study groups. The results were reported in the form of a dendrogram.

Statistica 10 (StatSoft Inc., USA) was used for the statistical analyses of data.

## Results

In this study, the concentrations of eight elements were measured in two groups: 31 patients with periodontitis and 29 healthy volunteers. The elements were divided into two groups: essential elements, Ca, Cu, Fe, Mg, Mn and Zn, and toxic metals, Cd and Pb.

### The Relationship Between Periodontal Disease and the Concentration of the Selected Metals in Oral Fluid and Blood

The concentrations of eight studied metals in the oral fluid and blood of periodontal patients and healthy subjects along with basic statistical parameters are given in Tables 1 and 2. Among the essential metals in the oral fluid of the patients, Ca revealed the highest mean levels at 39.2 mg L^−1^, followed by Mg (9.9 mg L^−1^) and Fe (1.0 mg L^−1^). The other metals were present at relatively lower levels. The toxic metals had low concentrations, with the highest being that for Pb (15.8 μg L^−1^). It is worth noting that Mn concentration in the oral fluid of patients (41.1 μg L^−1^) was considerably higher compared to the value reported in another publication [23], where the mean concentration of this element was 2.94 ± 2.83 μg L^−1^. On the other hand, a study of Al-Rawi and Talabani [24] presented similar results to ours (concentrations of Mn: 6.0 ± 3.0 μg L^−1^). It can be concluded that the concentration of Mn in the saliva varies widely. This is probably related to the effects of diet, to dietary supplements or to different places of residence.

**Table 1:** Basic statistical parameters for metals distribution in the oral fluid of patients and healthy volunteers

Elements	Patients (*n* = 31)					Healthy volunteers (*n* = 29)				
	Whole group			Men (*n* = 14)	Women (*n* = 17)	Whole group			Men (*n* = 10)	Women (*n* = 19)
	Mean ± SD	Range	Median	Mean ± SD	Mean ± SD	Mean ± SD	Range	Median	Mean ± SD	Mean ± SD
Ca (mg L^−1^)	39.2 ± 19.4	8.5–71.3	42.8	47.7 ± 25.1	38.5 ± 21.3	35.0 ± 18.4	14.8–75.7	29.8	22.2 ± 8.3	27.2 ± 7.1
Cd (μg L^−1^)	0.2 ± 0.1	<LOD–0.5	0.2	0.2 ± 0.1	0.2 ± 0.2	0.3 ± 0.3	0.04–1.0	0.2	0.5 ± 0.4	0.3 ± 0.3
Cu (μg L^−1^)	45.1 ± 5.0*	3.8–162.7	26.8	41.0 ± 44.0	48.4 ± 39.5	8.2 ± 5.2	1.1–20.6	8.2	8.1 ± 2.9	6.0 ± 5.1
Fe (mg L^−1^)	1.0 ± 0.6	0.4–2.7	0.8	0.9 ± 0.5	1.1 ± 0.8	0.9 ± 0.7	0.2–3.5	0.8	687.7 ± 310.9	767.4 ± 547.1
Mg (mg L^−1^)	9.9 ± 5.4*	1.2–19.9	9.2	11.5 ± 5.3	8.6 ± 5.3	5.2 ± 2.5	2.1–9.9	4.5	6.1 ± 3.0	4.1 ± 1.4
Mn (μg L^−1^)	41.1 ± 15.6*	<LOD–74.8	39.6	38.2 ± 17.3	29.3 ± 24.2	15.0 ± 8.2	0.5–32.3	16.6	15.0 ± 5.9	15.6 ± 7.5
Pb (μg L^−1^)	15.8 ± 8.2	7.7–35.4	13.0	16.2 ± 9.3	15.5 ± 7.3	12.4 ± 7.9	0.3–28.8	10.5	7.8 ± 2.6	10.0 ± 8.2
Zn (μg L^−1^)	79.1 ± 103.2	0.5–378.5	40.6	58.3 ± 60.8	104.9 ± 126.2	75.3 ± 74.4	0.3–124.3	57.3	67.0 ± 58.9	46.7 ± 32.1

*p < 0.05 with respect to the control group

**Table 2:** Basic statistical parameters for metals distribution in the blood of patients

Elements	Patients with periodontitis (*n* = 13)			Data for healthy people [21, 22]
	Mean ± SD	Range	Median	Mean ± SD
Ca (mg L^−1^)	87.0 ± 52.6	53.1–190.1	68.0	67.3 ± 14.3
Cd (μg L^−1^)	0.7 ± 0.5	0.2–1.5	0.5	0.8 ± 0.3
Cu (mg L^−1^)	0.6 ± 0.2	0.3–0.8	0.6	1.0 ± 0.2
Fe (mg L^−1^)	451.3 ± 43.1	377.8–499.4	466.5	427.3 ± 46.6
Mg (mg L^−1^)	37.1 ± 21.8	2.8–85.2	31.9	32.0 ± 3.0
Mn (μg L^−1^)	11.3 ± 6.6	<LOD-19.3	10.0	8.7 ± 2.4
Pb (μg L^−1^)	25.1 ± 19.5	6.5–46.7	19.0	41.5 ± 16.5
Zn (mg L^−1^)	2.2 ± 0.7	1.3–3.4	2.0	5.3 ± 1.0

Similarly to the group of patients, in the control group, Ca (35.0 mg L^−1^), Mg (5.2 mg L^−1^) and Fe (0.9 mg L^−1^) were present at the highest levels in saliva among the essential metals, while Pb (12.4 μg L^−1^) emerged as the major contributor among the toxic metals. The high standard deviations observed in the concentration of metals are probably due to the biological spread between individuals.

A two tailed Mann–Whitney test examination of the data showed that there were no significant differences between the levels of Ca, Cd, Fe, Pb and Zn in the saliva of periodontitis patients and healthy donors. However, the patients affected by periodontal disease showed statistically significant increases in the salivary concentrations of Cu (550 %), Mg (190 %) and Mn (273 %). The differences in salivary levels of these elements appeared to be related to periodontal disease.

The concentrations of the studied metals in the oral fluid were also evaluated for relative gender-based variations in both groups (Mann–Whitney test at *p* < 0.05). The average levels of Ca, Cd, Mg, Mn and Pb were higher in the oral fluid of male patients, while in the cases of Cu, Fe and Zn, the mean concentrations were found to be higher in the oral fluid of female (Table 1). In particular, the difference in Mg content between the two groups was quite considerable (8.6 ± 5.3 mg L^−1^ for women and 11.5 ± 5.3 mg L^−1^ for men). However, no statistically significant differences were observed in the oral fluid concentrations of the metals between women and men from the patients’ group. In the case of the control group, there were also no gender differences for metal concentrations (Table 1).

In the blood of periodontal patients, the highest concentrations of following essential elements, Fe (451.3 mg L^−1^), Ca (87.0 mg L^−1^), Mg (37.1 mg L^−1^), Zn (2.2 mg L^−1^) and Cu (0.6 mg L^−1^), were observed (mean values; Table 2). The other metals were present in trace amounts.

The measured concentrations were compared to the results for healthy people reported in other studies [21, 22]. The levels of Cd, Cu, Fe, Mg, Mn and Pb in the blood were similar in the examined groups, while the patients were characterized by considerably higher concentrations of Ca and Zn. This comparison is only to be used as an approximate reference and on statistical analysis can be applied to these data.

In the next step, the concentration of metals in the examined biological samples was compared in order to confirm whether oral fluid can be used as alternative material for the assessment of elemental status. Samples of blood and oral fluid were collected simultaneously from nine patients with periodontitis. The correlation coefficient of each pair of selected elements in these body fluids was calculated and it ranged from *r* = −0.413 (Zn) to *r* = 0.336 (Ca). The results suggested that there were no strong linear correlations between the concentrations of metals (*p* < 0.05). The mean concentrations of all elements, with the exception of Cd, were statistically significantly different from each other in the blood and the oral fluid (Mann–Whitney test, *p* < 0.05).

### Correlation Study

The metal-to-metal correlations in the oral fluid of periodontal patients and controls were investigated, and the results were presented in Tables 3 and 4. For patients affected by periodontitis, Ca was positively correlated with many metals: Pb (*r* = 0.752), Mg (*r* = 0.540), and Cd (*r* = 0.455). Other notable correlations were Fe–Mn (*r* = 0.499) and Pb–Cd (*r* = 0.400). A quite similar pattern of mutual relationships between the elements was observed in the case of the control group. Strong positive correlations were again found between Ca and three metals: Mg (*r* = 0.782), Pb (*r* = 0.652) and Zn (*r* = 0.540). Additionally, some significant correlations were also observed between Mg and two elements: Pb (*r* = 0.456) and Zn (*r* = 0.384).

**Table 3:** Correlation coefficient matrix of selected elements in the oral fluid of periodontal patients (*n* = 31)

	Ca	Cd	Cu	Fe	Mg	Mn	Pb	Zn
Ca	1.000	0.455*	0.134	0.005	0.540*	−0.252	0.752*	0.038
Cd		1.000	0.229	−0.073	0.097	0.041	0.400*	−0.321
Cu			1.000	−0.124	−0.122	0.050	0.280	0.236
Fe				1.000	−0.237	0.499*	0.111	0.115
Mg					1.000	−0.211	0.159	−0.152
Mn						1.000	−0.354	−0.208
Pb							1.000	0.246
Zn								1.000

*p < 0.05

**Table 4:** Correlation coefficient matrix of selected elements in the oral fluid of healthy donors (*n* = 29)

	Ca	Cd	Cu	Fe	Mg	Mn	Pb	Zn
Ca	1.000	−0.349	0.335	0.028	0.782*	−0.224	0.652*	0.540*
Cd		1.000	0.016	0.124	−0.263	−0.065	−0.236	0.026
Cu			1.000	0.353	0.289	0.177	0.113	0.325
Fe				1.000	−0.054	0.350	−0.123	−0.004
Mg					1.000	−0.130	0.456*	0.384*
Mn						1.000	−0.066	−0.207
Pb							1.000	0.320
Zn								1.000

*p < 0.05

**Fig. 1:**
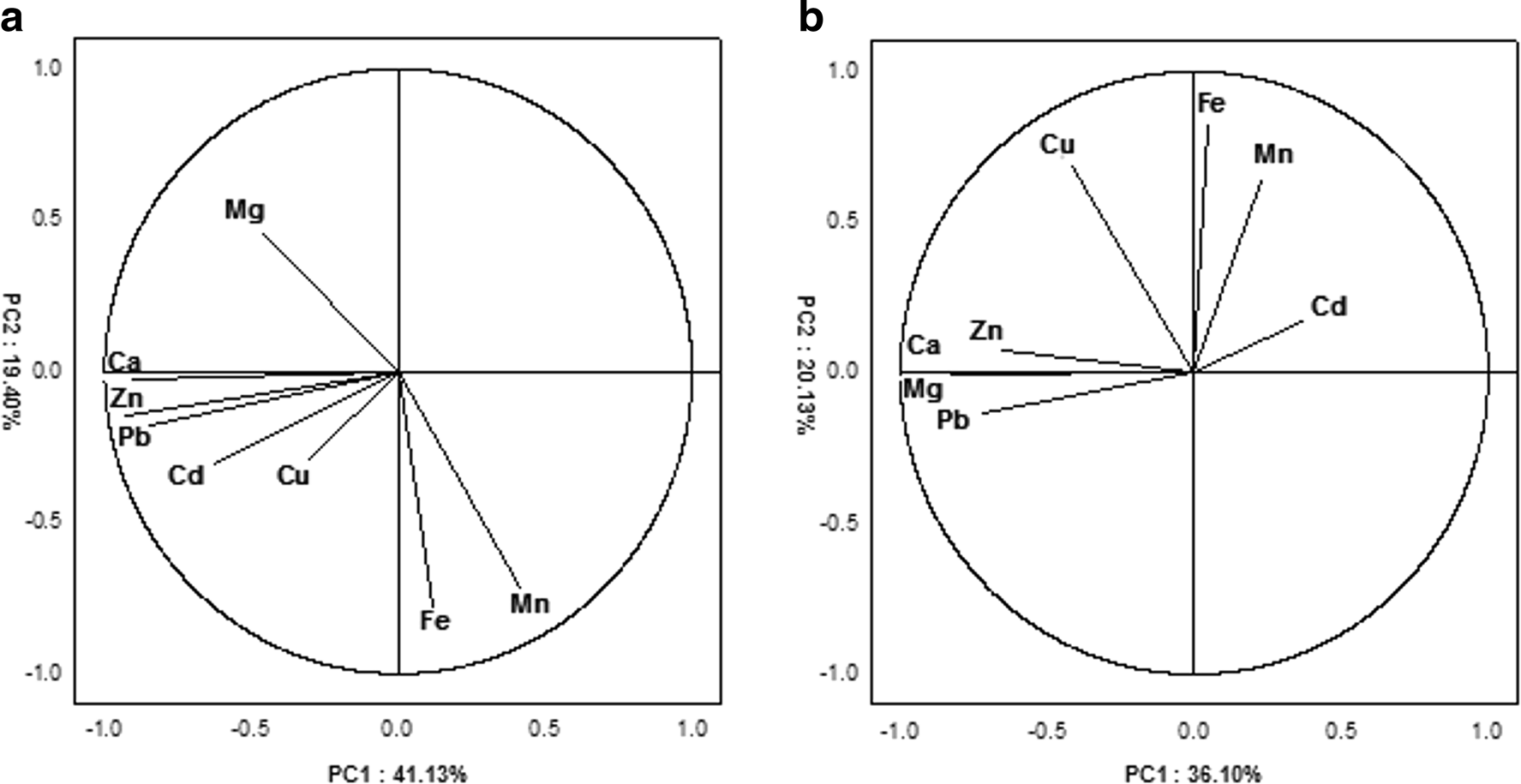
Projection of variables: the concentration of metals in the saliva of patients with periodontitis (**a**) and healthy volunteers (**b**), on the plane of two first principal components (PC1 and PC2)

Accordingly, principal component analysis was employed to visualize the interactions between selected metals in the oral fluid of the two groups. Figure 1 shows the score plots for the first two PCs (PC1–PC2) and was constructed from variables (the concentrations of elements in the oral fluid). The direction of vector indicates its correlations with another vector. The smaller angle between two vectors represents stronger correlations between the variables. In the group of periodontal patients (Fig. 1a), significant positive associations can be observed between Zn–Fe, Fe–Pb, Co–Ca, Co–Zn and Pb–Cr. Concentration of Cd, Mn and Mg in oral fluid was inversely associated with the rest of the elements. In the case of control group (Fig. 1b), strong positive correlations were found between the following elements, Ca–Mg, Zn–Co, Ca–Co, Ca–Zn and Pb–Mg, while Cr were negatively correlated with the majority of metals. A visual examination of Fig. 1 showed that the pattern of mutual dependence of metals in oral fluid of patients was different from those of healthy donors.

### Cluster Analysis

**Fig. 2:**
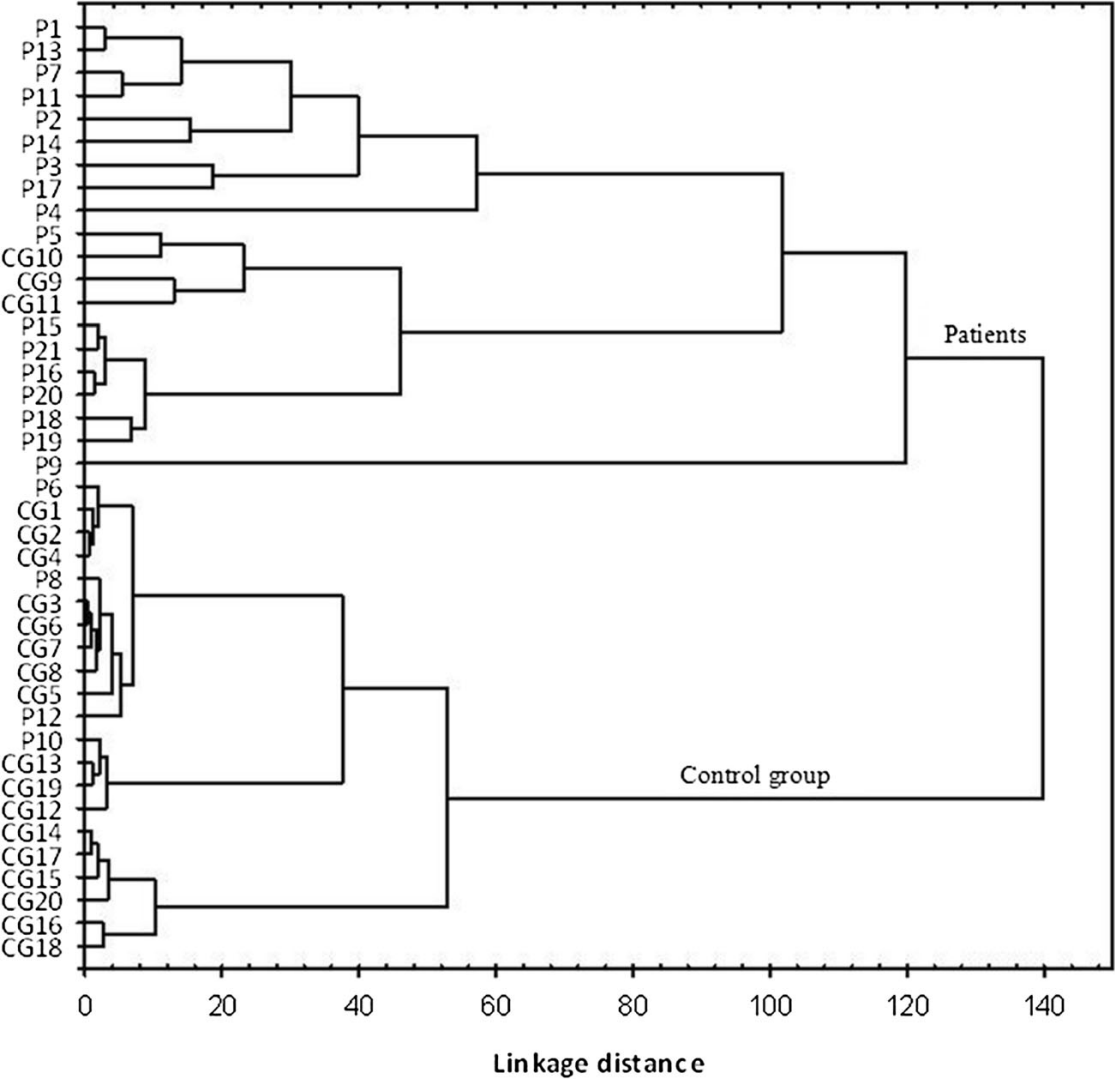
The dendrogram obtained for saliva representing cases from control group (CG1–CG20) and patients with periodontitis (P1–P20)

To examine whether it is possible to diagnose periodontal diseases on the basis of elemental content in oral fluid, hierarchical cluster analysis was performed. The results obtained are presented in the form of a clustering diagram in Fig. 2. It can be seen that the examined samples were divided into two groups at a linkage distance of 120. The top cluster contained periodontal patients (with three exceptions), while the second cluster had mostly healthy donors (with four exceptions). Considering the number of samples (40), it can be concluded that classification based on concentrations of eight metals in oral fluid gave satisfactory results.

## Discussion

Traditional clinical methods of periodontal disease diagnosis include the evaluation of several parameters (e.g. CAL = clinical attachment level, PD = probing depth) and radiographic measurements. They have some limitations, so many scientists try to find valid biomarkers which allow not only early detection of periodontal diseases but also evaluation of treatment effects [25, 26]. Recent examples of biomarkers are microRNAs (miRNAs) [26] and interleukin-1β (IL-1β) and prostaglandin E2 (PGE2) in saliva [27], or matrix metalloproteinase (MMP)-9 [28] and neutrophil gelatinase-associated lipocalin (LCN2) in gingival crevicular fluid [28].

Additionally, some studies have focused on a possible association between inorganic ions in oral fluid and periodontal disease. Khamees et al. [29] found that the levels of Na and Ca in patients with chronic periodontitis were significantly higher than in a control group, while no significant difference in the concentrations of K and Mg was found. Also there was a significant negative correlation between Na and the clinical attachment level (CAL) and a positive correlation between Ca and this periodontal parameter. In our study, there were no significant differences in the concentration of Ca between the periodontitis and control group but patients had a slightly elevated level of this metal. This agreed with results obtained by Sewón et al. [30] and Acharya et al. [31]. Authors concluded that the oral fluid concentration of Ca can be a risk factor for the development of periodontal diseases. Increased level of metal in saliva probably affects the mineralization of dental plaque and hence calculus formation [32]. Unremoved dental calculus can cause gingivitis that can further develop into periodontitis [19].

The results obtained in our laboratory for the concentration of Mg in oral fluid were in good agreement with the results reported by other authors [33, 34], who also found that patients with periodontitis had a higher oral fluid level of this metal than healthy people.

There have only been a few studies of the levels of elements other than main electrolytes (like Ca and Mg) in oral fluid. Mahmood et al. [34] measured the concentration of three oral fluid metals: Zn, Cu and Mg. They observed that the concentrations of Cu were significantly higher (*p* < 0.001), while Mg and Zn concentrations were lower (*p* < 0.001) in the group of patients than in controls. In our study, the periodontal group also had an increased concentration of all these metals in oral fluid (*p* < 0.05), but the differences were statistically significant for Cu and Mg.

Cluster analysis, due to a significantly different distribution of the eight elements in the oral fluid of those examined, enabled the correct division of the samples into two groups: patients and healthy subjects. Such a statistical approach has successfully been applied in distinguishing between drug-free people and drug abusers [35], the diagnosis of cancer [24] and Parkinson’s disease [36]. To our knowledge, there have been no reports of the classification of periodontal patients by cluster analysis based on metal concentrations in saliva.

The relationship between concentrations of metals (both in oral fluid and blood), and periodontal disease, as also the possibility of diagnosis on that basis, may in the future serve to prevent the development of such diseases, as well as to treat existing ones.

## Conclusion

The present study was aimed at exploring the changes in the content of metals in the oral fluid and blood of patients affected by periodontal disease. The paper also examined the use of cluster analysis in the classification of samples of saliva based on the concentration of selected metals.

Salivary metal levels varied over a relatively wide range. Most of the examined elements were detected at elevated concentrations in the saliva of periodontal patients. However, the differences were statistically significant (*p* < 0.05) in the case of three metals: Cu, Mg and Mn.

The correlation study revealed that there were noticeably different mutual dependences of the selected elements in the saliva of two groups: patients affected by periodontitis and the healthy controls. There were fewer correlations between metals in the saliva of patients than in the controls.

The results of cluster analysis indicate that the metal profiles of oral fluid in those with periodontal disease are different from the controls. The data obtained from this study can be a basis for the future development of diagnostic and prognostic biomarkers for periodontal disease.
